# Use of a digital tool to support the diagnostic process in memory clinics–a usability study

**DOI:** 10.1186/s13195-024-01433-8

**Published:** 2024-04-08

**Authors:** Aniek M. van Gils, Hanneke F. M. Rhodius-Meester, Dédé Handgraaf, Heleen M. A. Hendriksen, Astrid van Strien, Niki Schoonenboom, Annemieke Schipper, Mariska Kleijer, Annemiek Griffioen, Majon Muller, Antti Tolonen, Jyrki Lötjönen, Wiesje M. van der Flier, Leonie N. C. Visser

**Affiliations:** 1 Alzheimer Center Amsterdam, Neurology, Vrije Universiteit Amsterdam, Amsterdam UMC, Amsterdam, The Netherlands; 2 Amsterdam Neuroscience Neurodegeneration, Amsterdam, The Netherlands; 3 Department of Geriatric Medicine, The Memory Clinic, Oslo University Hospital, Oslo, Norway; 4 Department of Internal Medicine, Geriatric Medicine Section, Vrije Universiteit Amsterdam, Amsterdam UMC, Amsterdam, The Netherlands; 5 Amsterdam Cardiovascular Sciences, Amsterdam, The Netherlands; 6 Department of Geriatric medicine, Jeroen Bosch Ziekenhuis, Den Bosch, The Netherlands; 7 Department of Neurology, Spaarne Gasthuis, Haarlem, The Netherlands; 8 Department of Neurology, HagaZiekenhuis, location Zoetermeer, Zoetermeer, The Netherlands; 9 Combinostics Ltd, Tampere, Finland; 10 Department of Epidemiology and Data Sciences, Vrije Universiteit Amsterdam, Amsterdam UMC, Amsterdam, the Netherlands; 11 Department of Medical Psychology, Amsterdam UMC location University of Amsterdam/AMC, Amsterdam, The Netherlands; 12 Amsterdam Public Health, Quality of Care, Amsterdam, The Netherlands; 13 Division of Clinical Geriatrics, Center for Alzheimer Research, Department of Neurobiology, Care Sciences and Society, Karolinska Institutet, Stockholm, Sweden

**Keywords:** usability, implementation, memory clinic, digital tools, clinical decision support, innovations, communication

## Abstract

**Background:**

Both memory clinic professionals and patients see value in digital tools, yet these hardly find their way to clinical practice. We explored the usability of a digital tool to support the diagnostic work-up in daily memory clinic practice. We evaluated four modules that integrate multi-modal patient data (1.cognitive test; cCOG, and 2. MRI quantification; cMRI) into useful diagnostic information for clinicians (3. cDSI) and understandable and personalized information for patients (4. patient report).

**Methods:**

We conducted a mixed-methods study in five Dutch memory clinics. Fourteen clinicians (11 geriatric specialists/residents, two neurologists, one nurse practitioner) were invited to integrate the tool into routine care with 43 new memory clinic patients. We evaluated usability and user experiences through quantitative data from questionnaires (patients, care partners, clinicians), enriched with thematically analyzed qualitative data from interviews (clinicians).

**Results:**

We observed wide variation in tool use among clinicians. Our core findings were that clinicians: 1) were mainly positive about the patient report, since it contributes to patient-centered and personalized communication. This was endorsed by patients and care partners, who indicated that the patient report was useful and understandable and helped them to better understand their diagnosis, 2) considered the tool acceptable in addition to their own clinical competence, 3) indicated that the usefulness of the tool depended on the patient population and purpose of the diagnostic process, 4) addressed facilitators (ease of use, practice makes perfect) and barriers (high workload, lack of experience, data unavailability).

**Conclusion:**

This multicenter usability study revealed a willingness to adopt a digital tool to support the diagnostic process in memory clinics. Clinicians, patients, and care partners appreciated the personalized diagnostic report. More attention to education and training of clinicians is needed to utilize the full functionality of the tool and foster implementation in actual daily practice. These findings provide an important step towards a lasting adoption of digital tools in memory clinic practice.

**Supplementary Information:**

The online version contains supplementary material available at 10.1186/s13195-024-01433-8.

## Introduction

Due to the aging population, by 2050, the number of people with dementia is expected to double [1]. Moreover, when novel disease-modifying therapies (DMTs) become available, this will have an additional impact on patient flow to memory clinics [2]. For all these people, a timely and accurate diagnosis is important, as even without a cure for neurodegenerative disorders, a timely diagnosis and, thus, appropriate care influences the disease trajectory, improve quality of life, delay institutionalization, and aid in future planning [3, 4].

The number of diagnostic tests and biomarkers available to ensure a timely and accurate diagnosis of the underlying neurodegenerative disease causing dementia is increasing, and new assessment tools, including digital technologies, develop rapidly [3, 5, 6]. Diagnostic tests, including neuropsychological testing, magnetic resonance imaging (MRI), and biomarkers such as amyloid and tau in cerebrospinal fluid (CSF), can aid in differential diagnosis. However, guidelines for optimally interpreting tests are lacking, resulting in broad practice variation in applying and communicating diagnostic tests [7–9]. Smart solutions integrating available patient data can support clinicians with diagnostic challenges, facilitate comprehensive patient communication, and harmonize diagnostic approaches among memory clinics [10].

We previously developed and validated the PredictND tool [10], a web-based tool to support clinicians in differential diagnosis and patient communication. In a validation study in academic memory centers, the tool accurately classified dementia subtypes and increased clinicians’ confidence in the diagnosis [10]. The tool was later commercialized as the cNeuro® platform (Combinostics Oy, Finland). To address the clinical need for the ability to perform cognitive testing digitally from home and to support comprehensive patient communication, a new prototype digital tool has been developed. This tool is based on the cNeuro® platform and consists of four modules that integrate multimodal patient data (digital cognition; cCOG [11], and MRI quantification; cMRI [12]) into useful diagnostic information for clinicians (cDSI) and understandable and personalized information for patients (patient report) [13].

Despite the developments in digital tools and the positive attitudes of clinicians and patients regarding these advancements [14], their use is currently limited to highly specialized clinics. In contrast, particularly local clinics could benefit most from their use [15]. To translate evidence-supported interventions into practice and enhance their lasting adoption in local clinics, implementation research is crucial [16, 17]. Therefore, in the current study, we investigated the usability of the prototype digital tool when used by clinicians in daily memory clinic practice, to lay the foundation for future implementation and eventually support sustained adoption.

## Methods

### Study design

We performed an explanatory sequential mixed-methods study in which qualitative research follows quantitative results to aid in explaining or elaborating the data [18]. To operationalize this concept in our study, we enriched quantitative data from observations and questionnaires for clinicians and patients, with qualitative data from open-ended questions and in-depth interviews. The study was conducted from December 2021 to December 2022 among clinicians (i.e., physicians, as well as other staff members, such as nurse practitioners), patients, and care partners from five Dutch memory clinics identified and approached using the Dutch Memory Clinic network website (http://www.zorgvoorbeter.nl/geheugenpoli): the Center for Geriatric Medicine Amsterdam, Amsterdam UMC (COGA); Amstelland Hospital, Amstelveen; Spaarne Hospital, Haarlem; HagaHospital, Zoetermeer, and Jeroen Bosch Hospital, Den Bosch. We aimed for the inclusion of ten patients per memory clinic. Apart from excluding patients with insufficient knowledge of the Dutch language, there were no exclusion criteria to reflect the actual population in the memory clinic.

The study was approved by the VUmc Medical Ethical Committee and the local Medical Ethical Committee in all five centers. All patients provided written informed consent for their data to be used for research purposes.

### Digital tool

The digital tool consisted of four modules: (1) digital cognitive test tool (cCOG); (2) automated MRI-quantification tool (cMRI); (3) clinical decision support tool based on machine learning (cDSI) extended for supporting cCOG, and (4) diagnostic reporting, in which all information is summarized in an easily understandable overview for clinicians and patients (patient report). Although all modules are integrated, each can be used independently since the classifier can handle missing data. All modules are described in detail in Table 1.

**Table 1:** Overview of modules integrated in the digital tool: 1. cCOG, 2. cMRI, 3. cDSI, 4. Patient report

Module 1. Web-based cognitive testing, cCOG cCOG is a web-based cognitive test tool that was validated in CN, MCI, and patients with dementia [11]. cCOG has a completion time of approximately 20 minutes and consists of 6 subtasks (memory learning and recall, modified trail making test A/B, reaction time task, fragmented letter test) and 7 questions relating DLB’s core features [19].﻿ cCOG can be performed at home or in the clinic and is currently available in Dutch, English, Danish, Finnish, Swedish, and Italian.	
Module 2. Quantification of MRI images, cMRI cMRI extracts imaging markers from MRI scans using automatic quantification methods [20]. From T1 images, volumes of 133 brain regions and various disease-specific imaging biomarkers, such as computed medial temporal lobe (cMTA) score, computed global cortical atrophy (cGCA) score and anterior posterior score, are calculated. From FLAIR, white-matter hyperintensity volumes and computed Fazekas are quantified. MRI images acquired on either 1.5T or 3T scanners can be used [12]. cMRI is able to accurately aid in the differential diagnosis of the four main diseases causing dementia (AD, DLB, VaD, and FTD) [12, 20]	
Module 3. Clinical decision support tool based on machine learning, cDSI Clinical decision support tool based on machine learning (cDSI). The DSI is a data-driven classifier that provides a scalar index between zero and one, indicating a patient’s disease, being CN, AD dementia, VaD, DLB, or FTD [21, 22﻿]. cDSI considers all available patient data, including demographic information (age, sex, education), neuropsychological test results, cCOG results, cMRI quantification, and CSF results (amyloid-beta, t-tau, p-tau). The DSI has been extensively studied and was validated in clinical practice [10, 23﻿].	
Module 4. Patient report The available patient information is combined into a simple patient report which was co-created in a previous study with patients and care partners [13]. The patient report contains information about the diagnosis, individual test results, practical tips, and where to find more information. Clinicians can add their own text to subsections of the report. In the current study, clinicians had to fill in the final diagnosis themselves.	

*AD* Alzheimer’s disease: *CN* cognitively normal: *CSF* cerebrospinal fluid: *DLB* dementia with Lewy bodies: *FTD* Frontotemporal lobe dementia: *MCI* mild cognitive impairment: *p-tau* phosphorylated tau: *VaD* Vascular dementia

The digital platform runs in a standard web-browser using the Microsoft Azure cloud. All data transfers and storing are encrypted and anonymized. cMRI can be integrated into the organization’s PACS through a gateway (DICOM node). To complete the cCOG tests, patients received a link in an email. The test could then be performed via the Internet, on a tablet or computer, either at home or in the clinic.

### Study procedures

**Fig. 1:**
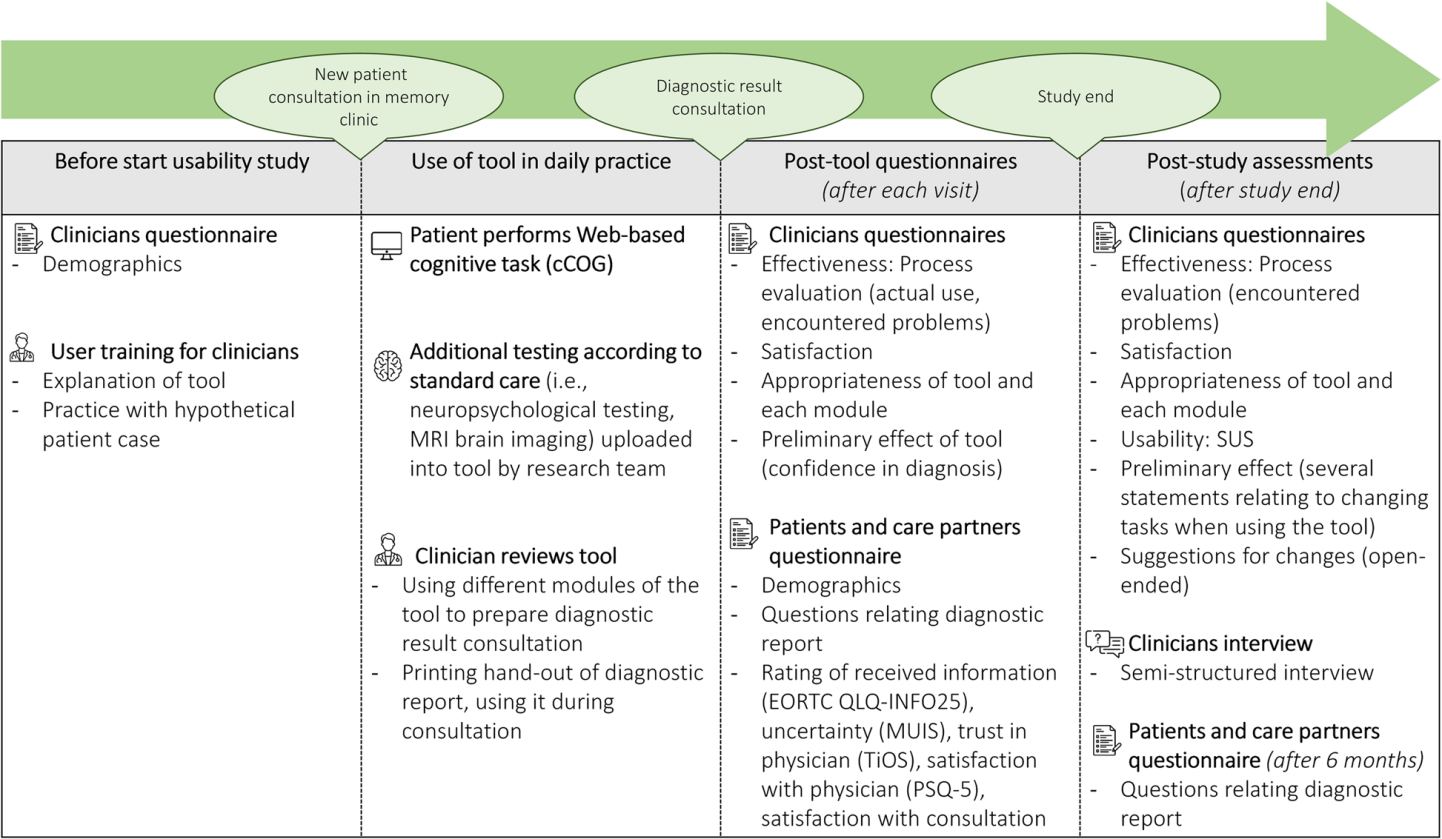
Study procedures of the usability study. EORTC QLQ-INFO25: European Organization for Research and Treatment of Cancer information Questionnaire [24], MUIS: Measurement of Uncertainty in Illness [25], TiOS: Trust in Oncologist Scale [26], PSQ-5: Patient Satisfaction Questionnaire Short form [27],﻿ SUS: system usability scale [28]

Figure 1 shows a schematic overview of the study procedures. First, participating clinicians completed an initial pre-study questionnaire covering demographic information. Then, they received user training from the research team. This training consisted of a face-to-face explanation of the modules and a practice session with a hypothetical patient case. Afterward, they received a printed manual. After the study started in the clinic, patients and their accompanying care partners were consecutively recruited after their first memory clinic visit. Patients were included by either a study team member (DH, SM, or AG), the clinician who had seen the patient, or a (research) nurse, depending on the staff availability at each clinic. Fourteen clinicians participated: neurologists (*n*=2), internal geriatric specialists (*n*=5), internal medicine residents (*n*=6), a nurse practitioner (*n*=1), and 43 patients and 28 care partners.

Next, patients were asked to perform the web-based test cCOG (module 1), during their memory clinic visit or at home. For those opting for remote testing, an email invitation with a link to the test was sent. Of the 43 patients, 38 (88%) patients completed cCOG, of whom 16 (42%) were remote and 22 (58%) were in the hospital. Additionally, patients received routine clinic-specific testing (i.e., neuropsychological testing and/or brain imaging). Thus, clinicians were not instructed to obtain a complete dataset for their patients. Instead, they were informed to provide standard care and use the available data in that naturalistic setting. The research team uploaded the available diagnostic test results into the tool to foster optimal integration into the clinical routine. Of note, the MRI protocols differed per clinic. In one clinic, the required T1 or FLAIR images were not acquired in the standard protocol, rendering uploading and analyzing scans impossible.

Following this, the clinicians were invited to review the different modules of the tool prior to each patient’s diagnostic result consultation. Particularly for the patient report, clinicians were encouraged to use it *during* the consultation and provide the patient with a printed hand-out. A study team member was present in each memory clinic to assist patients with performing cCOG and aid the clinicians in using the tool. After each diagnostic result consultation, clinicians were asked to complete a questionnaire about patient characteristics and their experience with the tool. Additionally, all patients and care partners received a questionnaire containing demographic information and questions relating to the patient report and satisfaction.

After the inclusion period (on average 18 weeks), clinicians received a questionnaire to assess the overall usability of the tool and were invited for an in-depth interview. Patients and care partners received a follow-up questionnaire six months after the consultation to assess the long-lasting effects of the patient report.

### Data collection

To assess the usability – defined as the extent to which representative individuals can use an innovation to achieve specific goals in a specific context [29] of the tool, we assessed various relevant constructs, such as *effectiveness* (f.e., encountered errors when using the tool), *efficiency (*f.e., time efficiency), and *satisfaction* (overall satisfaction, trust) [29]. In addition, we collected data on the *appropriateness* (usefulness of the tool/perceived fit) and *preliminary effect* of the tool [30], i.e., for clinicians, we gained data on confidence in the diagnosis after using the tool. For patients and care partners, we aimed to explore outcomes such as satisfaction with different aspects of the consultation and assessed their opinions about the patient report. Figure 1 shows an overview of the questionnaires used at different time points. See Additional file 1 for all questionnaires.

#### Data collection – clinicians

Before the study started, we collected demographic information of clinicians. Directly after each diagnostic result consultation, clinicians provided details about the patient’s diagnosis and evaluated the use of the tool using several questionnaires. In an open-ended question, we asked clinicians to comment or make suggestions for changes. At the end of the study, we further assessed the overall usability, again using questionnaires, and in an open-ended question, we asked clinicians to list the tool’s most positive and negative aspects and changes for continued use in daily practice. To enrich the questionnaire data, clinicians were invited to participate in a post-study interview, in which *n*=10 clinicians participated. A semi-structured interview guide was developed, aligning with the post-study questionnaire. The clinicians were asked to illustrate their answers to the questionnaire and elaborate on their user experiences. The interviews lasted 7-18 minutes and were held in person (*n*=4) or by telephone/video call (*n*=6) by one of the researchers (AG; medical doctor or DH; neuropsychologist). Interviews were audio recorded and transcribed verbatim.

#### Data collection - patients and care partners

Demographic information of the participating patients and care partners and their understanding of the diagnosis were collected via a questionnaire after the diagnostic result consultation. Furthermore, we assessed their rating of the received information, uncertainty, trust, and satisfaction using several validated questionnaires (Additional file 1). Additionally, we asked for their opinion about the patient report in an open-ended question. After six months, we repeated a subset of the questionnaire to reevaluate the patient report.

### Analyses

Participants’ characteristics and quantitative survey outcomes were summarized descriptively. Mean Likert scale scores were calculated for 5-point items. Mean SUS scores were calculated and curve-graded, centered at 68 (according to [31]). Data were analyzed with IBM SPSS Statistics version 28.0. We used thematic content analysis to analyze the transcripts of the interviews and open-ended responses [32]. One coder (AG: medical doctor) roughly organized the transcripts according to the main outcomes of effectiveness, efficiency, satisfaction, appropriateness, and preliminary efficacy. The transcripts were further categorized by inductively deriving new codes and forming an initial coding tree. Two coders (HRM: medical doctor, LV: psychologist) independently coded the interviews using this code tree. AG, HRM, and LV compared and discussed the codes intensively to identify overarching themes. The quantitative findings, such as the observed use of the tool modules and the reported SUS scores, were discussed against these themes and integrated, yielding a set of core findings. All authors reviewed and refined the core findings and supported them with quotes. MAXQDA 2022 (VERBI Software, 2021) [33] was used for the qualitative analysis.

## Results

Table 2 shows an overview of clinician characteristics and quantitative study outcomes. The clinicians (*n*=14) were, on average, 38±6 years old, almost all (91%) were female, and they had a mean of 7±7 years of experience in memory clinic care. Participants with ID 9 to ID 14 were residents in the final years of their residency training, with limited experience in memory clinic care. All other clinicians had at least four years of experience. All participating hospitals use electronic health records (EHRs), so all clinicians had some level of digital proficiency in advance.

**Table 2:** Overview of participating clinicians and quantitative study outcomes

ID	Age (y)	Specialization	Experience in MC (y)	Tool used (n)	Modules used			Satisfaction [0-100, mean]^1^	Usability, SUS [0-100, mean]^2^	Confidence in diagnosis [0-100, mean]^1^
					cCOG	cMRI	cDSI/ report			
1	49	Neurology	21	5	2	1	4	65 (20-80)	67.5	73 (55-90)
2	42	Geriatrics	16	4	2	0	4	28 (0-70)	35.0	90 (80-95)
3	51	Neurology	15	8	5	7	8	93 (90-100)	87.5	89 (80-95)
4	38	Geriatrics	11	4	4	0	4	51 (20-65)	70.0	85 (80-95)
5	39	Neurology	10	NA^3^	NA^3^	NA^3^	NA^3^	NA^3^	70.0	NA^3^
6	35	Internal elderly care medicine	6	4	3	1	3	53 (20-90)	27.5	77 (65-90)
7	42	Geriatrics	6	1	1	0	1	80	52.5	70
8	36	Geriatrics	4	1	0	0	1	68 (55-80)	52.5	78 (75-80)
9	36	Internal elderly care medicine	2	1	1	0	0	50	Missing	90
10	31	Internal elderly care medicine	2	1	0	0	1	42 (30-55)	67.5	60 (40-75)
11	30	Internal elderly care medicine	2	2	0	0	2	50	35.0	80
12	32	Internal elderly care medicine	1	1	0	0	1	80	60.0	65
13	39	Internal elderly care medicine	0	1	1	1	0	80	Missing	80
14	33	Internal elderly care medicine	0	1	0	0	1	70	Missing	80

^1^Post-tool questionnaires, average scores and range, if applicable, ^2^Post-study questionnaire, ^3^Not applicable: clinician 9 is a nurse practitioner who prepared the diagnostic results consultations for clinician 8, but did not take part in these consultations

*MC* memory clinic: *SUS* system usability scale. NOTE a SUS score ≥68 would be considered above average

**Fig. 2:**
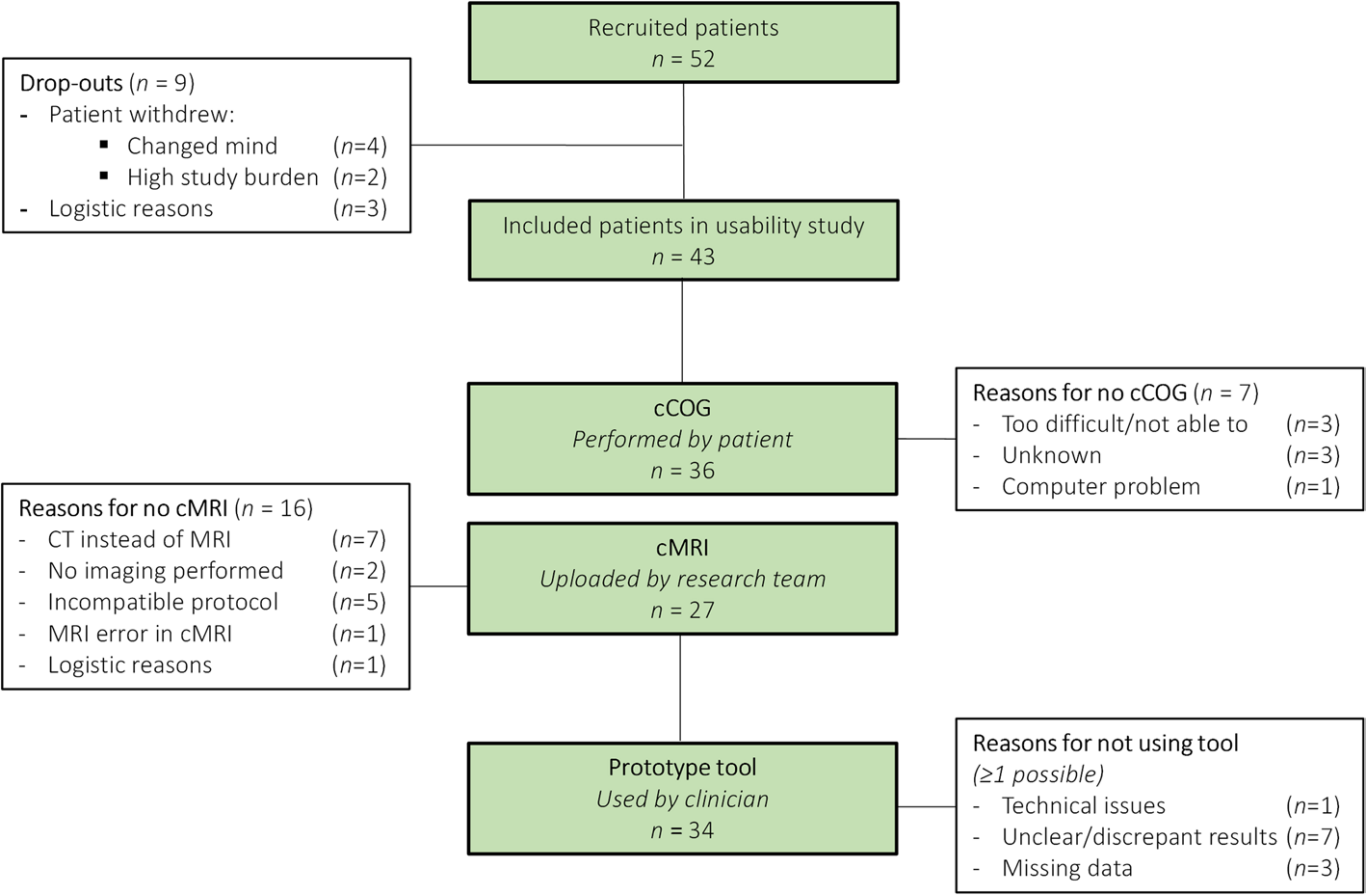
Study flowchart and process evaluation

As shown in Fig. 2, 52 patients were initially enrolled. Six patients withdrew during the study (because of high study burden or unspecified reasons), and three patients were excluded for logistic reasons (no diagnostic assessment at all or due to time constraints). In total, data from 43 patients, together with 28 accompanying care partners, were available for analysis. Data on the 6-months follow-up questionnaire were available for *n*=23 patients and *n*=15 care partners. Most of the patient follow-up questionnaires were missing because they were never received back despite of reminders (*n=*15*)*, patients were no longer able to fill in the questionnaire due to their cognitive decline (*n*=3), or had died (*n=2*). Table 3 shows the characteristics of the included patients and care partners.

**Table 3:** Characteristics of participating patients (*n*=43) and their care partners (*n*=28)

	Patients	Care partners
	*n*=43	*n*=28
**Demographic information** Age, mean	73±11	65±11
Sex, female	13 (28%)	20 (71%)
Education, years	9.7±4.4	10.2±4.0
**Clinical information**^1^ MMSE [0-30]	26±4	-
MoCA [0-30]	21±4	-
CDR [0-3]	0.7±0.5	-
**cCOG performed** (n, %)	38 (88%)	-
**Brain imaging, MRI**^1^ (n, %)	34 (79%)	-
**Syndrome diagnosis**^1^ Cognitively normal/SCD	7 (16%)	-
Mild cognitive impairment	19 (44%)	-
Dementia	14 (33%)	-
Postponed diagnosis	3 (7%)	-
**Etiology in case of dementia**^1^ Alzheimer’s disease	9 (64%)	-
Vascular dementia	2 (14%)	-
Other	3 (22%)	-
**Relation to patient** Spouse	-	16 (70%)
Son/daughter(in law)	-	4 (17%)
Other	-	3 (13%)
**Post-tool questionnaires**^2^ Uncertainty [1-5]^3^	2.9±1.4	2.8±1.2
Trust in physician [1-5]^4,5^	4.2±1.0	4.4±0.7
Satisfaction with physician [0-10]^4,5^	7.5±2.2	7.9±1.7
Satisfaction with information[1-4]^4,5^	2.9±0.8	3.1±0.8
Satisfaction with consultation in general [0-100]^4,5^	74±20	82±21

Data represent mean±SD or n(%). Missing data ranged from 49% (MMSE) to 19% (post-tool questionnaires)

^1^Data obtained from clinicians questionnaire

^2^Data on the post-tool questionnaire were missing for 5/43 patients because the tool was not used (*n*=1) or the questionnaire was never returned (*n*=4)

^3^Higher scores indicate more uncertainty

^4^Higher scores indicate more trust/satisfaction

^5^Average scores

*MMSE* Mini-mental state examination: *MoCA* Montreal Cognitive Assessment: *MRI* magnetic resonance imaging: *SCD* Subjective Cognitive Decline

Patients were, on average, 73±11 years old (range: 37-94) and predominantly male (*n*=30, 72%). Most patients (44%) were diagnosed with mild cognitive impairment (MCI) and one-third (33%) with dementia, mainly due to Alzheimer’s disease (64%). Most care partners were female (71%) and spouses. As shown in Table 3, patients and care partners were generally satisfied with their clinician and (the information provided during) the diagnostic result consultation.

Figure 2 shows the study flowchart of the observed use of the tool in daily practice. In 56% of the patients (24/43) the dataset was complete (i.e., containing both cCOG and cMRI results). The tool was used in 34 out of 43 patients (79%). Strikingly, clinicians used the tool in highly variable ways. Modules 3 and 4 (cDSI and patient report) were used most often (30/34, 88%). Many patients completed cCOG (88%), yet clinicians only used this module in 53% of patients prior to or during the diagnostic result consultation. Although the cMRI module was available for 27 patients, it was only used for ten patients by four clinicians. These numbers show a large variability in the actual use of the tool. Likewise, quantitative study outcomes (Table 2) are also highly variable and dependent on the actual use of the tool. As such, the qualitative findings are crucial for interpretation of the quantitative results. Our synthesis of quantitative and qualitative findings resulted in four core findings, which we describe below, illustrated with quotes and quantitative results.

### Clinicians highly valued the diagnostic report, since it contributes to patient-centered and personalized communication.

Clinicians expressed the wish to provide patients with printed or written personalized information but lack time to create such a personalized report in their daily practice. They indicated the tool’s diagnostic report helped them fulfill this need. Additionally, the clinicians noticed that their patients appreciated receiving the diagnostic report.

- *Quote 1.1.: “…so I think they [the patients] thought it was normal [to receive personalized information]. And I too, I think it is normal. I only wouldn’t know how to do this.” (Clinician 7)*
- *Quote 1.2.: “Yes, I actually think it is nice to share that on paper with all our patients, especially patients with dementia. We often share the patient letter, but that predominantly contains medical jargon. Yes, that is very patient-friendly and it really should be done, but it still takes quite a lot of time to do it properly. And, um, in reality that doesn’t happen very often." (Clinician 2)*

Concerning the potential beneficial effects, the clinicians mentioned that the tool created an overview, aiding effective patient communication. In the questionnaires, the majority (73%) of the clinicians (totally) agreed that communication with the patient becomes more efficient when using the tool.

- *Quote 1.3.: "since I struggle a lot with all the information I have to talk about during such a consultation, I think it really is good to provide the patient with some written text." (Clinician 11)*

In addition to supporting their communication with patients, clinicians highly valued the diagnostic report from the patient’s perspective because they consider it beneficial for patients’ and care partners’ understanding and recall of the information provided in the diagnostic result consultation.

- *Quote 1.4.: “Because patients only remember a very limited part (…) it is actually a bad news conversation that people receive when they are diagnosed with dementia and sometimes they only remember 5 to 10% of what the physician told them: they basically go home empty-handed. But now they will have an actual form with the results on which it is possible to type additional information (…) or a description of the follow-up process. Also, the patients can read back another time of ‘My diagnosis is Alzheimer's disease and that diagnosis was made based on memory tests and a scan’ or things like that. In short, I am really super enthusiastic about the whole information page..” (Clinician 4)*

They further indicated that the abovementioned pros of having a diagnostic report would outweigh the time investment needed to create such a report within the tool.

*Quote 1.5.: “yes, definitely, I certainly think there are a lot of positives. I didn’t think of it as extra work (…) no, not at all, I really thought it is a good thing. (…) No, I really think it pays off, and I therefore didn’t mind the extra work.” (Clinician 3)*

**Fig. 3:**
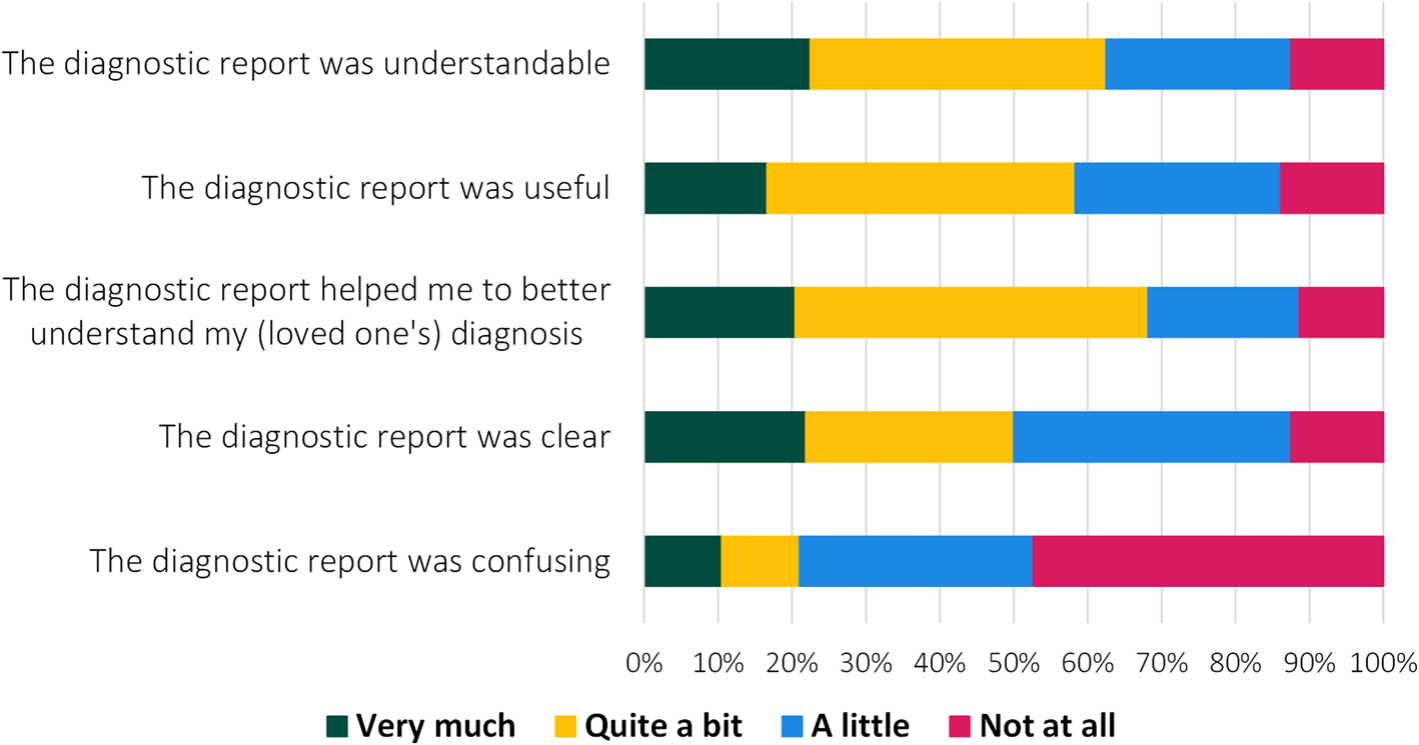
Patients’ and their care partners’ rating of the diagnostic report

The diagnostic report was also valued by patients and care partners, who, directly after the consultation, rated the diagnostic report as clear, understandable, and useful (Fig. 3). In the open-ended question about the diagnostic report, a patient positively highlighted the visual aspect of the diagnostic report:

*“All of the extra information, the images, the examinations, etc. are instructive and clarify a lot. This document complements the doctor's explanation. This document helps in understanding my illness, in addition to only hearing what the doctor has to say. It is also nice to see the information visualized.” (female, 73 years, dementia due to Alzheimer’s disease).*

A care partner underscored the understandability of the diagnostic report:

*“A good way to present difficult information (both emotional and medical) in a clear and understandable manner.” (44 years, daughter of patient with dementia).*

After six months, satisfaction with the diagnostic report (*scale 0-4*) was 3.0±0.5 for patients and 3.2±0.4 for care partners, and 78% of the patients and 83% of the care partners would want their doctor to use the diagnostic report again in a future consultation.

### Clinicians considered the tool satisfactory to varying degrees, especially in addition to their own clinical competence.

Satisfaction ratings with the tool overall varied among the clinicians, as shown in Table 2. In the interviews, the clinicians explained that the diagnostic process is difficult, and a tool that objectively interprets patient data can be helpful, as illustrated by quotes 2.1 – 2.2.

- *Quote 2.1.: (…) But there is the difference for me, that I have some kind of objective means that validates or challenges my clinical reasoning." (Clinician 2)*
- *Quote 2.2.: “yes, I understand the question very well, because cognitive diagnostics is indeed really difficult (…) I always tell the residents that I mastered many subjects in medicine quite quickly, but that it really took me a while to get the hang of cognitive diagnostics. (…) so it would be nice if it in some way could be made easier.” (Clinician 6)*

Furthermore, clinicians’ confidence in the diagnosis was slightly, though not significant (*P=0.23)*, higher when the tool was used (80±13) than when not used (74±11). Additionally, 46% of the clinicians indicated that using the tool increased their confidence. In the post-tool questionnaire, most clinicians (55%) agreed that the tool makes the differential diagnosis of neurodegenerative disorders easier. However, the same complexity of the diagnostic process also led clinicians to emphasize that the doctor’s interpretation is always needed to personalize the results provided by the tool:

- *Quote 2.3.: "I also notice that I can have slightly different conversations with patients with the same diagnosis simply because of differences in people’s characters and where the nuances are. I don’t think you can incorporate that very well in such a tool." (Clinician 12)*

Still, they also described how they could use the tool to double-check the results of their own clinical reasoning:

- *Quote 2.4: “Yes, and I can also imagine that one can use it as a kind of checker. That you think like ‘okay this is what I think it is, let's see what cNeuro thinks.’ If it thinks it is something completely different, maybe I should reconsider my diagnosis.” (Clinician 3)*

In its current state and level of integration, some clinicians questioned the added value of the tool in addition to their knowledge and expertise. Some clinicians specified that the tool did not (yet) incorporate some of the diagnostic tools used in their clinical practice (e.g., specific neuropsychological tests, but also brain scans such as computer tomography (CT) or positron emission tomography (PET)).

- *Quote 2.5.: “Yes, well, it may be even nicer if you can also include all the standard diagnostic tests in your analysis, in other words, to expand that tool even more to be able to enter everything that is being done. Yes, that would be ideal.” (Clinician 6)*
- *Quote 2.6.: “The tool made the diagnosis but I myself made the same diagnosis with the information that was available to me (…) So then obviously the question is ... does the tool really help you? Actually no, it is merely doing the same thing as I do with the clinical information I have.” (Clinician 4)*

Others did sometimes not trust the output because in their population, not all data were available and they therefore thought that they could not use the tool to its full potential, or they doubted the reliability of the outcome.

- *Quote 2.7.: (…) it is also somewhat related to the fact that I often didn’t agree that much with the results that were generated by the tool. I think this again was due to the incomplete datasets all those people had."(Clinician 10)*

### The appropriateness of the tool depends on the patient population and the specific purpose of the diagnostic process.

Although the clinicians considered the tool acceptable in addition to their clinical competence, they felt it would be most appropriate for specific patients or patient populations, depending on the purpose of the diagnostic process. As illustrated by quote 3.1, some clinicians especially valued the tool for a diagnostic process focused on determining the etiological diagnosis. Quote 3.2. illustrates that the tool could be used to decide whether to apply additional diagnostic tests.

- *Quote 3.1.: “Yes and at the same time I think (…) the idea of this tool that gives a kind of weighted result of does this fit AD, does this fit FTD, does this fit something else? That's actually quite useful of course.” (Clinician 3)*
- *Quote 3.2.: “Well, I think that with certain types of patients you could use it as a kind of pre-selection tool, to determine how far you have to go and what the probability is in advance (…) as part of a kind of two-stage package perhaps? (…) if you ensure that the test has good sensitivity and can filter out a part of those [persons with] subjective complaints of which you already know that they do not require further examination.” (Clinician 6)*

Since some clinicians focus more on a syndrome diagnosis rather than an etiological diagnosis to adequately organize care for their patients, they rated the tool as less useful for the patient population in their daily clinical practice. This was often mentioned by clinicians working with older individuals.

- *Quote 3.4.: “in such an aged population characterized by a mix of symptoms it often is more subtle: I think we not only diagnose a particular disease or asses the etiology, but also pay more attention to syndrome diagnosis and (…) what impact is it going to have on the patient’s daily life (…) what issues are you going to work on, being more practical than just appointing a case manager, in an early stage providing information/giving advice that you believe can be useful to the patient. With such an approach the etiology is often a little less important.” (Clinician 12)*

In addition, the availability of data for this patient population of older aged individuals was frequently limited. This was either because patients were unable to complete cCOG, or because MRI and cerebrospinal fluid (CSF) assessment were not part of standard care procedures in geriatric or internal medicine practices, as mentioned in this citation:

- *Quote 3.5.: “Most patients are not subjected to an LP or MRI, (…) usually the standard workup is sufficient for a proper diagnosis and I therefore think your tool is not very useful (…). I can just imagine if you puzzle a little bit; what is it now (…) Then I can imagine you can weigh the combination LP with MRI a little better” (Clinician 2)*

### Facilitators and barriers for using the tool in daily practice.

Clinicians evaluated the usability of the tool in the context of the current study (design), in which they were provided with a prototype version of the tool. They received a short training before the study started, which was elaborated during the usability study period based on feedback. Thus, some clinicians received more training than others. Using the tool in the context of the current usability study, the clinicians identified some facilitators and barriers to using the tool in daily practice.

### Facilitators for using the tool

In the post-study questionnaire, 7/11 clinicians (slightly) agreed that the tool could easily be integrated into their daily work process. Regarding factors that would facilitate clinicians using the tool, clinicians who had used the tool often (≥4 times), mentioned that they found it easy to use whereas clinicians who less often used the tool found it more difficult to use.

- *Quote 4.1.: "It is easy to use, you can log in very easily and then you can easily find the right patient and click further to see the patient details. ” (Clinician 4 – used the tool four times)*
- *Quote 4.2.: “For a software program I found it quite complicated, especially also because it uses a lot of abbreviations, so if you haven’t use it for a while then you have to remember what they stand for, so you click from one thing to the other (…).” (Clinician 7 – used the tool once)*

To facilitate adequate use of the tool, the clinicians also indicated that practice makes perfect, i.e., repeated and consistent use facilitated mastering how to optimally work with it.

- *Quote 4.3.: “(…) I have only had 2 patients that I've really used it with so then each time you still have to really get back in a little bit and I don't think it saved time, but I yes I can imagine if you switch easier or use it more often that it does at least not take longer.” (Clinician 12)*

### Barriers to using the tool

Some clinicians mentioned that the high workload and limited time in their clinics could hinder the process of learning to work with a new tool and, therefore, the appropriate use of such a tool.

- *Quote 4.4.: “As an option, but then I still have to um, you know then I would have to start using it (…) but then I thought, here is yet another software program that has to be used and there already is so little time to properly deal with your outpatient visits.” (Clinician 2)*

Another more practical barrier was that the tool was not integrated into the EHR hence they had to log in to a different website and they had to put in the data twice (once in the EHR and once in the tool).

- *Quote 4.5: “(…) the biggest job is putting in the raw scores from all those tests. (…) and I am afraid you should also integrate it into [the electronic health record].” (Clinician 10)*

## Discussion

In this usability study, we examined the actual use and user-experiences of a digital tool, also referred to as a clinical decision support system (CDSS), to support memory clinic clinicians in their daily practice of differential diagnosis and patient communication. The insights gained serve as an important step toward future implementation of such digital tools in clinical practice. In line with earlier survey findings [14], clinicians were willing to use the digital tool in clinical practice. Nonetheless, the large variability in actual use and views of clinicians highlight the important challenges of implementing digital tools in (memory clinic) practice.

The failure to translate effective interventions into routine practice has been recognized for many healthcare innovations [34, 35]. It has been estimated that it takes 17 years for clinical innovations to become available to patients after being proven efficacious [17]. In this context, usability testing is important because by understanding the users’ attitudes and needs, the innovation can be adapted and the time to implementation reduced [36]. Therefore, we evaluated the usability from the perspectives of clinicians as the intended end-users, by assessing effectiveness, efficiency, and satisfaction both quantitatively and qualitatively and have learned important lessons that we describe below.

First, almost all clinicians indicated that the patient report meets a need to provide patients with written or printed (personalized) diagnostic information, and patients and care partners underscored this need and, thus, the added value of the tool. In a landscape of increasing numbers of diagnostic tests and with DMTs on the horizon [6], using the personalized patient report can aid in creating an overview for both clinicians and patients and allow better comprehension of diagnostic test results, the diagnosis and its implications [13]. Furthermore, a personalized report contributes to a patient-centered approach, which benefits patients by increasing patient satisfaction, quality of life, and relation with their physician [37]. With all these benefits and the positive attitude of clinicians in mind, implementation efforts should be aimed at the patient report first. To stimulate its adoption, minimizing the disruption of clinicians’ daily practice is important [38], making it essential to generate the report automatically to not (further) overburden clinicians. Ideally, the tool should be embedded in the EHR so that data can be automatically extracted and the report compiled accordingly. Until this is achieved, having a researcher/nurse who can generate the report is crucial to stimulate its use by clinicians.

Second, we learned that different clinicians face different questions regarding their patients with cognitive complaints and that the tool should aid in finding an answer to each of these questions. For certain clinicians and with certain patients, determining a syndrome diagnosis is crucial to organize appropriate care, while for others, an accurate etiological diagnosis holds greater importance. The clinicians rated the tool’s usability in light of their most urgent clinical question. Our findings indicated that the tool was rated less usable for the purpose of determining a syndrome diagnosis, while especially in a population of individuals of older age, this is often the clinically most relevant question. This makes sense because the primary focus of the current version of the tool is on etiological diagnosis [10, 22]. To encourage the adoption of the tool for all clinicians, it should be adapted to serve both clinical questions of syndrome and etiological diagnosis. Moreover, we anticipate a future in which the tool can assist in determining a patient’s eligibility for DMT, but for which certain adjustments need to be made. If the tool is adapted to address a broader range of clinical questions, clinicians indicated to be interested in using it for cross-checking their own clinical thoughts with the tool’s result or as an aid for triage and decision-making regarding which diagnostic test to perform next in a given patient. We are currently studying how the tool can guide stepwise diagnostic decision-making.

Third, the digital cognitive test module was less frequently adopted by clinicians, potentially because they did not perceive the added value of such a module since standard neuropsychological tests were already administered as part of their normal diagnostic routine. In addition, this module is a newly developed test that has to be CE-marked and is thus not yet approved for clinical practice[11]. Nonetheless, the digital cognitive test appeared to be usable since many patients were able to complete the test successfully. In an era of growing patient numbers, digital cognitive testing holds considerable value because it allows for cost-effective testing through self-administration [39] and can serve several purposes: as a screening tool, monitor disease progression during follow-up visits after diagnosis, and to measure treatment response [40]. To take advantage of these benefits, optimization of its use in clinical practice is warranted. To this end, it is important to prioritize feasibility studies and focus on user experiences while ensuring the validity and reliability of these digital cognitive tests [39].

Fourth, clinicians emphasized the importance of repetitive use to learn how to work with the tool. Such extensive familiarization was often not achieved in our study, due to clinicians’ high workload and limited education and training. Our findings thus highlight the significance of providing dedicated education and training. Previous studies also endorsed the importance of adequate training, revealing its significant influence on the success and efficiency of technologies [41, 42]. To successfully implement any digital tool into healthcare practice, it is imperative that clinicians are prepared through educational activities for digital tools in general. This can be achieved by teaching clinicians to use digital technology early on as a valuable addition to their own knowledge and skills, for instance, by integrating digital tools education into the medical curriculum [43]. Teaching clinicians how to adequately and effectively use a digital tool in clinical practice might save time and increase effectiveness in the end. In addition, tool-specific, onsite training should be offered, adapted for each memory clinic, as working methods and patient journeys often differ. Besides providing sufficient training, it is important to provide continuous technical support, peer-to-peer collaboration, written guidelines, and instructions to facilitate optimal implementation [41].

In the evolving dementia care landscape, digital tools will inevitably play their part [6]. Digital tools offer the potential to keep the patient journey accessible, harmonized, and patient-friendly [39]. A CDSS allows the funneling of patients to the memory clinic and selecting the right diagnostic workup, and digital cognitive testing enables cognitive testing in the comfort of one’s home [6]. In addition, there is an expected deficit in the number of dementia specialists [44], highlighting the need to establish effective and efficient digital tools in memory clinics. With this in mind, it was promising to learn that some clinicians did not (yet) see the added value of the tool since they found the tool could do roughly the same as they could do themselves. This could imply that other healthcare professionals, also the less experienced or less specialized, can be involved in diagnosing rather straightforward cases with the assistance of a tool [39]. In this way, employing the tool can save scarce specialist time for challenging diagnostic scenarios, for instance, when the tool indicates that a clear diagnosis cannot be made or the clinical question is complex.

### Strengths and limitations

One of the strengths of our study is that it was conducted in five different, local, non-academic memory clinics and involved clinicians with different specializations (neurology, internal geriatric medicine, clinical geriatric medicine) and broad-ranging experience resembling actual practice in the Netherlands. Because we applied no exclusion criteria, we included a real-life everyday sample consisting even of the most elderly individuals. However, we only included Dutch speaking patients and care partners, rendering generalizability to a non-Dutch speaking population. Despite the strengths, our study also had some limitations. Because of the usability study design, we included a relatively low number of clinicians and patients. Half of the clinicians used the tool only for one patient case and they tested/evaluated often only one module, meaning that their feedback is based on very limited experience. Nonetheless, the mixed-methods design allowed us to conduct an in-depth assessment of relevant usability outcomes. By performing this study, we gained first insight in actual use of a digital tool in daily memory clinic practice and identified some practical barriers, such as different MRI protocols that did not meet the requirements for image quantification. Therefore, this usability study can serve as a starting point for more in-depth studies aiming at implementing digital tools in memory clinics, such as the upcoming European PROMINENT study [45]. Furthermore, the tool holds the potential to be future-proof in that it can be extended by incorporating other data, such as the extended cCOG version for DLB [19] and amyloid PET [46], as well as FDG-PET and DaT-scan modules currently under development. These improvements can increase the accuracy and usefulness of the tool, not only for differential diagnosis but also for detecting patients eligible for DMTs. It remains to be elucidated how these advancements will affect the performance of the tool.

## Conclusion

In a changing dementia care landscape and an evolving patient journey, digital tools will play an inevitable part. Digital tools have the potential to allow easier and more accurate diagnosis, harmonization of care among memory clinics, and bridging a gap in providing personalized information provision. Nonetheless, implementation in clinical practice remains challenging. Our usability study provides a stepping stone for future implementation efforts and emphasizes the importance of dedicated education and training to prepare healthcare providers for the future use of digital tools alongside their clinical expertise.

### Supplementary Information

**Supplementary material 1.**

## Data Availability

The data acquired during the current study is available from the corresponding author on reasonable request.

## Abbreviations

*AD*: Alzheimer's disease
*CDSS*: Clinical decision support system
*COGA*: Center for Geriatric Medicine Amsterdam
*CSF*: Cerebrospinal fluid
*CN*: Cognitively normal
*DMT*: Disease-modifying therapy
*DLB*: Dementia with Lewy bodies
*DSI*: Disease state index
*EORTC QLQ-INFO25*: European Organization for Research and Treatment of Cancer information Questionnaire
*EHR*: Electronic health record
*FLAIR*: Fluid-attenuated inversion recovery
*FTD*: Frontotemporal dementia
*MC*: Memory clinic
*MRI*: Magnetic resonance imaging
MUIS: Measurement of Uncertainty in Illness
*p-tau*: Phosphorylated tau
PSQ-5: Patient Satisfaction Questionnaire Short form
*SCD*: Subjective cognitive decline
SUS: System usability scale
*TMT-A/B*: Trail Making Test A and B
*t-tau*: Total tau
*VaD*: Vascular dementia
