# Supplementary material for: Use of a digital tool to support the diagnostic process in memory clinics–a usability study
Source: Alzheimers Res Ther. 2024 Apr 8;16:75. doi: 10.1186/s13195-024-01433-8 (PMC11003066; doi:10.1186/s13195-024-01433-8)
Supplement: Supplementary file 1 — Supplementary material 1. [file 13195_2024_1433_MOESM1_ESM.docx]

Questionnaires usability study

# Questionnaire 1. Clinicians demographics

1. **What is your age**………………..
2. **What is your gender?**

- Male
- Female

1. **What is your function?**

- Medical specialist
- Physician, in medical specialist training
- Physician, *not* in medical specialist training
- Other, i.e………………..

1. **What is your specialization?**

- Neurology
- Clinical geriatric medicine
- Internal (geriatric) medicine
- Nursing home physician
- Psychiatry
- Other, i.e………………..

1. **If medical specialist: in which year did you complete your specialist training?** ….
2. **If in training: how far on are you in your specialist training?**....
3. **What type of institution do you work in?**

- Academic/university hospital
- Non-academic teaching hospital
- Non-academic, non-teaching hospital
- Nursing home
- Mental health service
- Other, i.e.……………………………

1. **How many years’ experience do you have in the diagnosis of patients in a memory clinic?** ………………..
2. **On average, how many new patients do you see per month in the memory clinic?** ………………..

*The following questions are about your own experience using digital tools.*

1. **Do you have experience using digital tools in the outpatient clinic?**

- Yes (multiple options possible)
  - Electronic patient files
  - In my daily practice I use a digital tool, namely………………..
  - I have experience using a digital tool in research, namely:……………….
  - Other, i.e………………..
- No

# Questionnaire 2. Clinicians: post-tool questionnaire

1. **Research number patient:** ………………..
2. **Date of first visit to memory clinic:** ………………..
3. **Date of diagnostic disclosure consultation:** ………………..
4. **Age of patient:** ………………..
5. **Gender of patient:**
6. **Sex of patient:**

- Male
- Female

1. **Score on Mini-Mental State examination MMSE:** ../30
2. **Score on Montreal Cognitive Assessment MoCA:** ../30
3. **Clinical dementia rating**: .../3
4. **Was brain imaging performed?**

- Yes, CT-brain
- Yes, MRI-brain
- No

1. **CT or MRI date:** dd/mm/yyyy
2. **Diagnosis**

| **Baseline diagnosis*:** | **Tick the box** |
| --- | --- |
| Cognitively normal/subjective cognitive complaints |  |
| Mild cognitive impairment (MCI) |  |
| Dementia |  |
| Postponed diagnosis |  |

| **If the diagnoses is dementia, what is the most probable etiology*?** | **Tick the box** |
| --- | --- |
| Alzheimer’s disease |  |
| Vascular dementia |  |
| Dementia with Lewy Bodies |  |
| Behavrioural variant frontotemporal lobe dementia |  |
| Semantic variant frontotemporal lobe dementia |  |
| Progressive non-fluent aphasia |  |
| Parkinson’s dementia |  |
| Creutzfeldt-Yescob’s disease |  |
| Corticobasal degeneration |  |
| Progressive supranuclear palsy |  |
| Amyotrophic lateral sclerosis |  |
| Dementia of unknown cause |  |
| Other, i.e.…………………………… |  |
| * According to the diagnostic criteria | |

1. **Is there a mixed dementia diagnosis?**

- Yes, namely………………………………………..
- No

1. **Here you can provide an explanation of the diagnosis:**

…………………………………………………………………………………………………………………………………………………………………………………………………………………………

1. **How high is your confidence in the diagnosis?**

| **Confidence in diagnosis** | **Tick the box** |
| --- | --- |
| High |  |
| Intermediate |  |
| Low |  |

1. **Confidence in the diagnosis on a VAS scale from 0-100**

| Lowest possible confidence | | | | | Highest possible confidence | | | | | | |
| --- | --- | --- | --- | --- | --- | --- | --- | --- | --- | --- | --- |
| **0** | **10** | **20** | **30** | **40** | | **50** | **60** | **70** | **80** | **90** | **100** |

**The following questions consider using the computer tool with this specific patient.**

1. **Did you use the tool during the consultation in which you discussed the diagnosis?**

- Yes
- No

1. **If not, why not?**

- Time restraints
- Technical issues
- Results of tool were unclear
- Patient or care partner refused
- I was not able to use the tool with this specific patient, because of………………..
- Other, i.e………………..

1. **If yes, which module(s) did you use?**

- 1. Cognitive test module (cCOG)
- 2. Imaging module (cMRI)
- 3. cDSI and fingerprint
- 4. Diagnostic report

1. **If yes, score the following 10 statements with one of five responses that range from strongly agree (5) to strongly disagree (1)**

| **Item** | **1** | **2** | **3** | **4** | **5** | **N/A** |
| --- | --- | --- | --- | --- | --- | --- |
| 1. Overall, the complete tool was useful for this specific patient | □ | □ | □ | □ | □ | □ |
| 1. The cognitive test module (cCOG) was useful | □ | □ | □ | □ | □ | □ |
| 1. The imaging module (cMRI) was useful | □ | □ | □ | □ | □ | □ |
| 1. The diagnostic report was useful | □ | □ | □ | □ | □ | □ |
| 1. Using the tool has strengthened my confidence in the diagnosis | □ | □ | □ | □ | □ | □ |
| 1. Using the tool made me doubt the diagnosis | □ | □ | □ | □ | □ | □ |
| 1. Using the tool decreased my doubts about the diagnosis | □ | □ | □ | □ | □ | □ |
| 1. Using the tool influenced the follow-up procedure* | □ | □ | □ | □ | □ | □ |
| 1. In general, I am satisfied with using the tool in this patient. | □ | □ | □ | □ | □ | □ |
| 1. The tool has given me new information/insights/ideas compared to what I already knew. | □ | □ | □ | □ | □ | □ |

*If you agreed with this statement: can you indicate how the tool has changed the follow-up procedure?……………………………………………………………………………………………………………………………………………………………………………………………………………

| **Lowest possible satisfaction** | | | | |  | | | **Highest possible satisfaction** | | | | |
| --- | --- | --- | --- | --- | --- | --- | --- | --- | --- | --- | --- | --- |
| 0 | 10 | 20 | 30 | 40 | | 50 | 60 | | 70 | 80 | 90 | 100 |

1. **Satisfaction with the tool on a VAS scale from 0-100**
2. **Here is space for suggestions or comments**

…………………………………………………………………………………………………………………………………………………………………………………………………………………………

# Questionnaire 3. Clinicians: post-study questionnaire

1. **Tool Usability Scale (SUS)**Score the following 10 items with one of five responses that range from strongly disagree (1) to strongly agree (5)

| **Item** | **1** | **2** | **3** | **4** | **5** |
| --- | --- | --- | --- | --- | --- |
| I think that I would like to use this tool frequently. | □ | □ | □ | □ | □ |
| I found the tool unnecessarily complex. | □ | □ | □ | □ | □ |
| I thought the tool was easy to use. | □ | □ | □ | □ | □ |
| I think that I would need the support of a technical person to be able to use this tool. | □ | □ | □ | □ | □ |
| I found the various functions in this tool were well integrated | □ | □ | □ | □ | □ |
| I thought there was too much inconsistency in this tool. | □ | □ | □ | □ | □ |
| I would imagine that most people would learn to use this tool very quickly. | □ | □ | □ | □ | □ |
| I found the tool very cumbersome to use. | □ | □ | □ | □ | □ |
| I felt very confident using the tool. | □ | □ | □ | □ | □ |
| I needed to learn a lot of things before I could get going with this tool. | □ | □ | □ | □ | □ |

1. **List the tool’s most positive aspect(s**):…………………………………………………………….
2. **List the tool’s most negative aspect(s**):……………………………………….………………….
3. **How would using this tool change various tasks?**

| I**tem** | **Strongly agree** | **Slightly agree** | **As much agree as disagree** | **Slightly disagree** | **Strongly disagree** |
| --- | --- | --- | --- | --- | --- |
| Patient data management becomes more efficient | □ | □ | □ | □ | □ |
| Communication and collaboration with colleagues becomes more efficient | □ | □ | □ | □ | □ |
| Communication with the patient becomes more efficient | □ | □ | □ | □ | □ |
| The tool makes predicting a neurodegenerative disorder easier. | □ | □ | □ | □ | □ |
| The tool makes the differential diagnosis of neurodegenerative disorders easier. | □ | □ | □ | □ | □ |
| Patient data management becomes more efficient | □ | □ | □ | □ | □ |
| Another task, namely… | | | | | |

1. **Did you experience any of the following issues while using the tool?**

| **Item** | **Not at all** | **Slightly** | **Neutral** | **A bit** | **A lot** |
| --- | --- | --- | --- | --- | --- |
| 1. Technical issues | □ | □ | □ | □ | □ |
| 2. Issues in the communication with the patient | □ | □ | □ | □ | □ |
| 3. Troubles using the tool | □ | □ | □ | □ | □ |
| 4. Troubles interpreting the results | □ | □ | □ | □ | □ |
| 5. Other issues, namely… | | | | |  |

1. **To what extent do the following statements apply to you?**

| I**tem** | **Strongly agree** | **Slightly agree** | **As much agree as disagree** | **Slightly disagree** | **Strongly disagree** |
| --- | --- | --- | --- | --- | --- |
| The cognitive test module (cCOG) was useful | □ | □ | □ | □ | □ |
| The imaging module (cMRI) was useful | □ | □ | □ | □ | □ |
| The diagnostic report was useful | □ | □ | □ | □ | □ |
| In the future, I would like to use a tool like this in my daily work. | □ | □ | □ | □ | □ |
| The tool can easily be integrated into my daily work. | □ | □ | □ | □ | □ |

1. **What would you like to change about the tool to continue using it in your daily practice?**

............................................................................................................................................................................................................................................................................................................................

## Patients and care partners questionnaire 1: post-tool questionnaire

1. **With whom did you complete this questionnaire?**

- No one
- Spouse
- Son/daughter
- Brother/sister
- Other family member
- Relative
- Other, namely…

1. **What is your age?** ………………..
2. **What is you gender?**

- Male
- Female

1. **What is the highest level of education that you have completed?**

- Primary education
- Lower secondary vocational education
- Lower general secondary education
- Higher general secondary education
- Pre-university education
- Senior secondary vocational education
- Higher professional education
- University education
- Other type of education, i.e…

1. **(For care partner) What is your relationship to the person who is experiencing symptoms or has dementia? I am…**

- Husband/wife/life partner
- Sister/Sister-in-law/Brother/Brother-in-law
- Daughter/Daughter-in-law/Son/Son-in-law
- Other, namely………………..

1. **What was the date of the consultation with your doctor?** ………………..
2. **On what date did you complete this questionnaire?** ………………..
3. **What was your diagnosis/the diagnosis of your loved one?**

…………………………………………………………………………………………………………………………………………………………………………………………………………………………

1. **Which follow-up appointments have been made?**

…………………………………………………………………………………………………………………………………………………………………………………………………………………………

1. **Below are a number of statements about your experiences with the complaints so far. Please read each statement carefully and circle the answer for each statement that best reflects the extent to which you agree or disagree with the statement.**

| I**tem** | **Totally agree** | **Slightly agree** | **As much agree as disagree** | **Slightly disagree** | **Totally disagree** |
| --- | --- | --- | --- | --- | --- |
| 1. I have a lot of questions without answers. | □ | □ | □ | □ | □ |
| 1. The purpose of the tests is clear to me. | □ | □ | □ | □ | □ |
| 1. I understand everything explained to me. | □ | □ | □ | □ | □ |
| 1. It is not clear what is going to happen to me. | □ | □ | □ | □ | □ |
| 1. The doctor gives me so much information that I don't know what is most important. | □ | □ | □ | □ | □ |
| 1. I don’t know what is wrong with me. | □ | □ | □ | □ | □ |
| 1. The explanations they give me about my condition seem hazy to me. | □ | □ | □ | □ | □ |
| 1. The doctor say things to me that could have many meanings. | □ | □ | □ | □ | □ |

1. **We are interested in the information you have received about aspects of your disease and its treatment, in order to improve your health care. Please answer ALL the questions yourself by circling the number that best applies to you. There are no right or wrong answers. The information that you provide will remain strictly confidential.**

*During your current disease or treatment, how much information have you received on:*

| I**tem** | **Not at all** | **A little** | **Quite a bit** | **Very much** |
| --- | --- | --- | --- | --- |
| 1. The diagnosis of your disease? | □ | □ | □ | □ |
| 1. The results of the medical tests you have already received? | □ | □ | □ | □ |
| 1. Are you satisfied with the amount of information you received? | □ | □ | □ | □ |
| 1. Did you wish to receive more information? | Yes | No |  |  |
| If yes, please specify on which topics? |  |  |  |  |
| 1. Did you wish to receive less information? | Yes | No |  |  |
| If yes, please specify on which topics? |  |  |  |  |
| 1. Overall has the information you have received been helpful? |  |  |  |  |

**11. Below are a number of questions related to the consultation you just had. You can answer these questions by placing a cross on the line next to the question where it best represents your answer.**

*For example:*

*Did you feel the doctor had enough time for you?*

*not at all*

*very much*

χ

**Looking back on the conversation with the doctor…**

To what extent did the doctor meet your needs or wishes?

*not at all*

*very much*

How actively did you get involved during the consultation?

*not at all*

*very much*

How satisfied are you with the information you received during this consultation?

*not at all*

*very much*

How satisfied are you with the (emotional) support that you received during this consultation?

*not at all*

*very much*

How satisfied are you in general about this conversation?

*not at all*

*very much*

1. **The questions below are about the confidence you have in your doctor after the consultation. Please read each statement carefully and circle the answer for each statement that best reflects the extent to which you agree or disagree with the statement.**

| I**tem** | **Totally agree** | **Slightly agree** | **As much agree as disagree** | **Slightly disagree** | **Totally disagree** |
| --- | --- | --- | --- | --- | --- |
| 1. Your doctor is totally honest in telling you about all the different treatment options available for your condition | □ | □ | □ | □ | □ |
| 1. You think your doctor can handle any medical situation, even a very serious one | □ | □ | □ | □ | □ |
| 1. Your doctor listens with care and concern to all the problems you have | □ | □ | □ | □ | □ |
| 1. Your doctor will do whatever it takes to get you all the care you need | □ | □ | □ | □ | □ |
| 1. All in all, you have complete trust in your doctor | □ | □ | □ | □ | □ |

**The following questions are specifically about the computer tool/results page that the doctor may have used during the results discussion.**

1. **Did your doctor show you the computer tool during the consultation?**

- Yes
- No

1. **If yes, which part of the tool did the doctor show you?**
   - Images of my brain scan
   - The report page in which my diagnosis and test results are summarized.
2. **If the doctor showed you the images of your brain scan: to what extend did you think this was**

| I**tem** | **Not at all** | **A little** | **Quite a bit** | **Very much** |
| --- | --- | --- | --- | --- |
| Understandable | □ | □ | □ | □ |
| Convenient | □ | □ | □ | □ |
| Confusing | □ | □ | □ | □ |
| Clear | □ | □ | □ | □ |

1. **To what extent did viewing the images help you (better) understand your diagnosis/the diagnosis of your loved one?**

- Not at all
- A little
- Quite a bit
- Very much

1. **If the doctor showed you the diagnostic report: to what extend did you think this was**….

| I**tem** | **Not at all** | **A little** | **Quite a bit** | **Very much** |
| --- | --- | --- | --- | --- |
| Understandable | □ | □ | □ | □ |
| Convenient | □ | □ | □ | □ |
| Confusing | □ | □ | □ | □ |
| Clear | □ | □ | □ | □ |

1. **To what extent did viewing the diagnostic report help you (better) understand your diagnosis/the diagnosis of your loved one?**

- Not at all
- A little
- Quite a bit
- Very much

1. **Did you miss information on the diagnostic report?**

- Yes
- No

1. **If yes, please indicate what information you have missed**:
   ………………………………………………………………………………………………………………………………………………………………………………………………………………

| **Very unlikely** | | | | |  | | | **Very likely** | | | | |
| --- | --- | --- | --- | --- | --- | --- | --- | --- | --- | --- | --- | --- |
| 0 | 10 | 20 | 30 | 40 | | 50 | 60 | | 70 | 80 | 90 | 100 |

1. **How likely are you to recommend this report page to another patient, on a scale from 0 to 100**
2. **Could you indicate why or why not?**
   ………………………………………………………………………………………………………………………………………………………………………………………………………………
3. **Did you receive a printed version of the diagnostic report?**

- Yes
- No

1. **If yes, for what purpose do you think you will use the report?**

- Help me understand my diagnosis/the diagnosis of my loved one
- To discuss with my spouse/childeren
- To show my family/friends
- To discuss with my general pracicioner
- For nothing/I do not yet know

| **Lowest possible satisfaction** | | | | |  | | | **Highest possible satisfaction** | | | | |
| --- | --- | --- | --- | --- | --- | --- | --- | --- | --- | --- | --- | --- |
| 0 | 10 | 20 | 30 | 40 | | 50 | 60 | | 70 | 80 | 90 | 100 |

1. **Overall, how satisfied are you with this appointment on a VAS scale from 0-100?**

## Patients and care partners questionnaire 2: After six months

1. **With whom did you complete this questionnaire?**

- No one
- Spouse
- Son/daughter
- Brother/sister
- Other family member
- Relative
- Other, namely…

1. **What has been the course of your memory/cognitive complaints the past six months?**

- Worsened
- Stable
- Improved
- Variable

1. **Has your diagnosis been changed by your doctor in the past six months?**

- Worsened
- Stable
- Improved
- Variable

1. **We are interested in the information you have received about aspects of your disease and its treatment, in order to improve your health care. Please answer ALL the questions yourself by circling the number that best applies to you. There are no right or wrong answers. The information that you provide will remain strictly confidential.**

| I**tem** | **Not at all** | **A little** | **Quite a bit** | **Very much** |
| --- | --- | --- | --- | --- |
| 1. The diagnosis of your disease? | □ | □ | □ | □ |
| 1. The results of the medical tests you have already received? | □ | □ | □ | □ |
| 1. Are you satisfied with the amount of information you received? | □ | □ | □ | □ |
| 1. Did you wish to receive more information? | Yes | No |  |  |
| If yes, please specify on which topics? |  |  |  |  |
| 1. Did you wish to receive less information? | Yes | No |  |  |
| If yes, please specify on which topics? |  |  |  |  |
| 1. Overall has the information you have received been helpful? |  |  |  |  |

1. **Below are a number of questions related to the consultation you just had. You can answer these questions by placing a cross on the line next to the question where it best represents your answer.**

*For example:*

*Did you feel the doctor had enough time for you?*

*not at all*

*very much*

χ

**Looking back on the conversation with the doctor…**

To what extent did the doctor meet your needs or wishes?

*not at all*

*very much*

How actively did you get involved during the consultation?

*not at all*

*very much*

How satisfied are you with the information you received during this consultation?

*not at all*

*very much*

How satisfied are you with the (emotional) support that you received during this consultation?

*not at all*

*very much*

How satisfied are you in general about this conversation?

*not at all*

*very much*

1. **Did your doctor show you the diagnostic report during the consultation?**

- Yes
- No

1. **Did you receive a printed version of the diagnostic report?**

- Yes
- No

1. **For what purpose did you use the report?**

- Consulted it again
- Showed to my family what is wrong with me
- Used to find more information about my diagnosis
- Nothing
- Other, namely………………..

1. **To what extent are you satisfied with the information on the diagnostic report?**

| Item | **Not at all** | **A little** | **Quite a bit** | **Very much** |
| --- | --- | --- | --- | --- |
| Are you satisfied with the information about your diagnosis? | **□** | **□** | **□** | **□** |
| Are you satisfied with the information about your test results? | **□** | **□** | **□** | **□** |
| Are you satisfied with the other information? | **□** | **□** | **□** | **□** |

1. **Do you have any suggestions, questions or remarks about the (information) on the report page?**………………………………………………………………………………………………………………………………………………………………………………………………………………
2. **If you were to come back after six months, would you like it if the doctor used the diagnostic report again?**

- Yes
- No

| **Lowest possible satisfaction** | | | | |  | | | **Highest possible satisfaction** | | | | |
| --- | --- | --- | --- | --- | --- | --- | --- | --- | --- | --- | --- | --- |
| 0 | 10 | 20 | 30 | 40 | | 50 | 60 | | 70 | 80 | 90 | 100 |

1. **Overall, how satisfied are you looking back on the diagnostic result consultation?**
